# Adapting tree-based multiple imputation methods for multilevel data? A simulation study

**DOI:** 10.3758/s13428-026-03008-x

**Published:** 2026-05-13

**Authors:** Nico Föge, Jakob Schwerter, Ketevan Gurtskaia, Markus Pauly, Philipp Doebler

**Affiliations:** 1 Department of Mathematics, Otto-von-Guericke Universität Magdeburg, Magdeburg, Germany; 2 Department of Statistics, TU Dortmund University, Dortmund, Germany; 3 Center for Research on Education and School Development, TU Dortmund University, Dortmund, Germany; 4 Research Center for Trustworthy Data Science and Security, UA Ruhr, Ruhr, Germany

**Keywords:** Multiple imputation, Multilevel data, Bias, Hierarchical data, MICE, MissRanger, Mixgb, Power, Type I error

## Abstract

When data have a hierarchical structure, such as students nested within classrooms, ignoring dependencies between observations can compromise the validity of imputation procedures. Standard (tree-based) imputation methods implicitly assume independence between observations, limiting their applicability in multilevel data settings. Although multivariate imputation by chained equations (MICE) is widely used for hierarchical data, it has limitations, including sensitivity to model specification and computational complexity. Alternative tree-based approaches have shown promise for individual-level data, but remain largely unexplored for hierarchical contexts. In this simulation study, we systematically evaluate the performance of novel tree-based methods—chained random forests (missRanger) and extreme gradient boosting (mixgb)—explicitly adapted for multilevel data by incorporating dummy variables indicating cluster membership. We compare these tree-based methods and their adapted versions with traditional MICE imputation in terms of coefficient estimation bias, type I error rates, and statistical power under different cluster sizes (25 and 50), missingness mechanisms (missing completely at random [MCAR], missing at random [MAR]), and missingness rates (10%, 30%, 50%), using both random intercept and random slope data generation models. The results show that MICE provides robust and accurate inference for level 2 variables, especially at low missingness rates (10%). However, the adapted boosting approach (mixgb with cluster dummies) consistently outperforms other methods for level 1 variables at higher missingness rates (30%, 50%). For level 2 variables, while MICE retains better power at moderate missingness (30%), adapted boosting becomes superior at high missingness (50%), regardless of the missingness mechanism or cluster size. These findings highlight the potential of appropriately adapted tree-based imputation methods as effective alternatives to conventional MICE in multilevel data analyses.

**Supplementary Information:**

The online version contains supplementary material available at 10.3758/s13428-026-03008-x.

## Introduction

Multiple imputation (MI) is a prominent and widely used technique for dealing with missing data. MI involves generating multiple plausible complete versions of the dataset to reflect the uncertainty about the missing values. Each dataset is then analyzed separately using standard statistical methods, and the results are pooled to obtain estimates and standard errors that account for both within- and between-imputation variability. While these steps are essential components of MI, “proper” imputations additionally require that uncertainty about the missing values is adequately propagated within the imputation model itself (Carpenter et al., 2023; Rubin, 1987, 1996). MI is particularly useful when the missingness mechanism is missing at random or missing completely at random (Little & Rubin, 2002; Schafer, 2000).

Ensuring that imputation models preserve the underlying relationships in the data and account for the missing data mechanism is crucial (van Buuren & Groothuis-Oudshoorn, 2011). This is a particular challenge in complex hierarchical datasets with multiple levels. Examples include clustering at individual and higher-level units like classes or schools. Preserving key multilevel features—such as contextual effects of level 1 variables, varying effects across clusters, and level 2 predictors—is crucial for valid inference, as failure to maintain the hierarchical structure can lead to biased analyses; however, this is frequently neglected in imputation (van Buuren, 2011).

Although fully conditional specification (FCS) or multivariate imputation by chained equations (MICE) is a prevalent approach in the social sciences, it presents several limitations, including its considerable complexity due to the challenges in the specification of the imputation model and computational intensity (van Buuren & Groothuis-Oudshoorn, 2011). Since MICE relies heavily on model specifications, it can lead to issues like overfitting and convergence errors, especially when dealing with multicollinearity and other instability problems (van Buuren & Oudshoorn, 1999).

Exploring alternatives to MICE, such as nonparametric tree-based methods, offers the potential for handling missing data in hierarchical structures, particularly in settings where their flexibility may be advantageous (Deng & Lumley, 2024; Stekhoven & Bühlmann, 2012). However, their performance in multilevel contexts—especially under linear mixed model assumptions common in the social sciences—remains largely unexplored.

Therefore, this simulation study addresses the following *research question*: Do tree-based imputation methods exhibit similar performance in terms of bias, type I error, and power as the standard level 2 imputation method? The specific tree-based methods are chained random forests (Stekhoven & Bühlmann, 2012; Tang & Ishwaran, 2017) and extreme gradient boosting with trees as base learners (Deng, 2023). To account for the multilevel data structure, we use dummy variables for each cluster, adapting tree-based imputation methods in the same way that Lüdtke et al. (2017) did for non-tree-based methods.

Although many different MI methods have been proposed and investigated for this setting—including multilevel MI with joint modeling, multilevel MI with fully conditional specifications, multilevel substantive-model-compatible MI with sequential modeling, or model-based treatment with Bayesian estimation, as recently reviewed by Grund et al. (2024)—tree-based methods have not been evaluated for multilevel data.

Our simulation study evaluated tree-based methods in a controlled setting using linear mixed models with linear effects and parametric data-generating processes. After that, missingness was introduced, and the resulting datasets were imputed using different methods. The imputed datasets were analyzed with a linear mixed model to evaluate type I error rates, power, standard deviation, and coefficient bias, thereby assessing the impact of imputation on subsequent multilevel analysis. This setup allows a direct comparison with MICE and deliberately focuses on linear multilevel scenarios. In particular, the data-generating process aligns with the assumptions of the linear mixed model used for analysis and does not include additional multilevel features such as contextual (between-cluster) effects of level 1 predictors, decomposed within- and between-cluster relations, or cross-level interactions to focus on a well-defined linear multilevel setting. While linear model-based imputation approaches are naturally well suited for settings with linear functional forms, tree-based methods are known to perform particularly well in the presence of nonlinearities and interaction effects, which can be more difficult to approximate using parametric models (Breiman, 2001; Chen & Guestrin, 2016). Accordingly, the present study focuses on linear functional forms to assess the performance of tree-based imputation methods in scenarios that align with standard linear multilevel analyses.

Readers should therefore interpret our findings with these limitations in mind: our results are most relevant to linear multilevel scenarios, while the advantages of tree-based approaches may become more apparent in nonlinear or interaction-heavy contexts that were not included in our study.

The remainder of the paper is organized as follows: In the next section, we briefly review multilevel data and existing (parametric) MI approaches for multilevel data structures. Next, the simulation study is detailed, starting with a review of the imputation approaches and an explanation of the simulated missingness mechanisms. We present simulation results in the following section with the help of various plots for the relative performance, and finally, we close with remarks on the current study and potential future work.

### Multilevel data

Multilevel data structures are common in social and behavioral sciences research (Hox & Roberts, 2010). This is often seen, for example, in educational research where students (level 1) are nested within classes, schools, or regions (level 2). Variation of higher-level variables can significantly influence the outcome variable. This calls for robust analysis methods that account for the complexity introduced by these hierarchical structures (Grund et al., 2024; Steenbergen & Jones, 2002), since simply ignoring the dependencies or aggregating everything to a single level can be deceptive (Aitkin & Longford, 1986; Grund et al., 2024). To maintain statistical integrity, it is crucial to employ appropriate imputation methods tailored for multilevel data. These methods should consider both within-cluster and between-cluster variability for a more accurate representation of the underlying data (Audigier et al., 2018; Grund et al., 2024; van Buuren, 2011).

Multilevel data often involve within- and between-cluster effects, where level 1 variables (e.g., student test scores) may have distinct effects at the individual level within clusters and contextual effects at the cluster level (McNeish et al., 2016). Additionally, random slopes allow the effects of level 1 variables to vary across clusters, capturing heterogeneity in relationships. Level 2 variables, such as cluster characteristics (e.g., class size or teacher experience), further influence outcomes and must be accurately modeled to preserve hierarchical relationships. Ignoring these features and the nested structure in imputation can lead to biased parameter estimates and incorrect variance components, particularly in multilevel MI, where preserving these relationships is essential for valid inference (Aitkin & Longford, 1986; McNeish et al., 2016; van Buuren, 2011). Hierarchical linear models, including random intercept and random slope models, provide a sophisticated statistical framework for analyzing such data structures, ensuring that both fixed and random effects are appropriately captured (Hox, 1998; Hox & Roberts, 2010).

### Existing imputation approaches for multilevel data

For a valid analysis, the imputation model must account for the dependency between observations inherent in the multilevel structure. Otherwise, inferences might be biased even when the statistical methods are otherwise appropriate (Audigier et al., 2018; Hox & Roberts, 2010). The bias of random-effect variance estimates as well as global fixed-effects confidence intervals depends on the cluster size, the relation of within- and between-cluster variance, and the missing data mechanism (Speidel, Drechsler, & Sakshaug, 2018). Two common imputation frameworks for multilevel data are joint modeling (JM) and fully conditional specification (FCS). In JM, imputations are generated simultaneously from a joint multivariate distribution for all variables (Hox & Roberts, 2010). In contrast, FCS imputes missing values iteratively on a variable-by-variable basis, using univariate conditional models based on other variables (van Buuren, 2011). MICE uses the conditional imputation approach from FCS and chains the variables by iteratively imputing one variable at a time based on the others (van Buuren & Groothuis-Oudshoorn, 2011). In this study, we use multivariate imputation by chained equations (MICE), implemented via the mice package, as a specific application of FCS, employing 2l.norm for level 1 variables and 2lonly.pmm for level 2 variables (van Buuren & Groothuis-Oudshoorn, 2011). This terminology reflects MICE as our chosen FCS implementation, with no aspects applying exclusively to FCS or MICE. A study from Enders et al. (2016) compared two imputation frameworks for multilevel data: JM and chained equation imputation (MICE with a two-level normal model 2l.norm). The joint model turned out to be better for analyses postulating distinct within- and between-cluster relations, while chained equations imputation turned out to be superior in random slope analysis (Enders et al., 2016).

For clustered data, both of these methods are effective in the broad context of *random intercept* models, even with variables at higher levels, as simulation results by Grund et al. (2018b) indicate. However, for *random slopes*, FCS appears to be more flexible than JM, but still has some limitations and is not all that reliable when data in an explanatory variable are missing (Grund et al., 2018b). Similar results were shown by Enders et al. (2016) for *random intercepts*. Additionally, Grund et al. (2018a) show that both approaches (FCS and JM) provide useful tools for dealing with missing data at level 2 in most applications in practice (especially for balanced data).

Recent studies highlight limitations of conventional multilevel MI approaches, such as FCS and JM, particularly for random-slope models. When explanatory variables have missing values, FCS can produce biased random slope estimates due to misspecification of conditional models (Erler et al., 2017; Erler et al., 2016). Model-based methods, such as substantive-model-compatible MI and Bayesian approaches, align imputation and analysis models more closely, improving accuracy for random slopes and complex hierarchical structures (Enders, 2023; Enders et al., 2020; Grund et al., 2021). These methods better capture within- and between-cluster effects in the presence of random slopes.

One advantage of choosing FCS over JM is that FCS grants more flexibility in creating multilevel models by splitting a *k*-dimensional problem into *k* one-dimensional problems. That is, for each of the variables with missing data, a regression model with a univariate outcome conditional on the other *k* − 1 variables is constructed. Furthermore, it is easier to avoid logical inconsistencies in the imputed data and incorporate methods to preserve unique features in the data (van Buuren et al., 2006); for example, temporal dependency can be taken into account in longitudinal data.

### Multiple imputation methods

In this simulation study, we implemented three main imputation methods: As baseline, we use multivariate imputation by chained equations (MICE) via the mice function from the R package of the same name (van Buuren & Groothuis-Oudshoorn, 2011). For level 1 variables, the level 1 normal model (2l.norm) is used, and for level 2 variables the level 2 class predictive mean matching (2lonly.pmm) is employed. Additionally, we include recent tree-based imputation methods: a fast implementation of imputation via random forests from the missRanger package (Mayer, 2021), and MI via gradient boosting implemented in the mixgb package (Deng, 2023). For MICE, the imputation methods and the prediction matrix for each variable are carefully adjusted to take into account the multilevel data structure. Two factors were varied for missRanger: We run the missRanger algorithm both with and without predictive mean matching (using 5 donors)^1^ and the variant (standard or an adapted implementation of missRanger). The adapted variant respects the multilevel structure of the data by including dummy variables for the respective cluster, while the standard implementation does not. In line with Drechsler (2015), who shows that multilevel models at the imputation stage can provide superior inference—especially for random effects—we acknowledge that running such models can be computationally demanding and that researchers often resort to simpler, dummy-based approaches. Indeed, representing cluster membership via dummies biases random-effects estimates more than fixed-effects estimates, yet remains popular because it is straightforward to implement with many conventional imputation tools. As we pioneer the adaption of tree-based methods to hierarchical data, we follow Drechsler’s rationale and incorporate dummy variables for clusters in our adjusted version of missRanger. While this does not capture the complete random-effects structure, it delivers a manageable, first-tier solution for incorporating level 2 distinctions without the overhead of fully specified multilevel modeling. Similarly, mixgb was adapted with additional dummy variables. We also used five imputations.^2^ For the pooling, Rubin’s rules were applied. Details of each method are given in the supplement.

### Multivariate imputation by chained equations

*Multivariate imputation by chained equations* (MICE) is a flexible and efficient imputation method that can treat missingness in a wide range of data types and analysis models. Its applications can be found in diverse research fields, including medicine, epidemiology, psychology, management, politics, and sociology (van Buuren & Groothuis-Oudshoorn, 2011).

MICE operates by iteratively drawing missing values from univariate conditional distributions given the other observed features. By repeatedly updating these imputations over several iterations, the method approximates the underlying conditional distribution, not unlike the burn-in phase of a Markov chain. The goal is not to produce increasingly accurate imputations but to generate “proper” imputations that reflect the uncertainty about the missing values and the parameters of the underlying model (Carpenter et al., 2023; Rubin, 1987). Once the iterative process stabilizes, multiple imputations are generated. The variability between imputations arises from independent draws from this stable conditional distribution (Kistner et al., 2024; van Buuren & Groothuis-Oudshoorn, 2011).

Imputing at level 2 requires additional considerations. One possibility is to have a separate model for level 2 variables, for example, a regression model that includes other level 2 variables as well as cluster-level components (e.g., mean, median) of the level 1 variables. As described by Grund et al. (2018a), imputations at level 2 are generated based on a probability distribution that considers multiple factors: aggregated information from level 1 (such as group means of relevant variables), the observed values of the other variables within the cluster, and specific model parameters.

As mentioned, mice is an R package for chained equation imputations using a function of the same name. To impute level 1 missing values, 2l.norm^3^ is used, which uses univariate missing data imputation with a two-level normal model. For level 2 variables, a level 2 class predictive mean matching (2lonly.pmm) is applied. Although Enders et al. (2016) used 2lonly.norm, we use 2lonly.pmm because 2lonly.norm follows the normality assumption, which does not hold in our case because we transformed some variables to aggregate them at level 2. 2lonly.pmm is a semi-parametric method, which is why it works in a wider range of cases.

### Chained random forest imputation

The idea of the random forest is to combine predictions from multiple decision trees to improve accuracy and reduce overfitting in both classification and regression tasks. In a random forest, each tree independently makes a prediction, and the final result is based on the majority vote (in classification) or the average prediction (in regression) of all the trees. Random forest can handle mixed types of data, is capable of addressing interactions and nonlinearity, and is robust to overfitting (Breiman, 2001). Because of its flexibility, random forest can also be adapted to imputation tasks (Golino & Gomes, 2016; Stekhoven & Bühlmann, 2012), transferring useful properties such as the lack of assumptions about normality or homoscedasticity.

As for MICE, the chained random forest imputation also starts with an initial guess that replaces all missing values in the data. The initial guess can be made by mean (default for metric data), median, mode (default for categorical data), or any other univariate imputation method. At this point, every variable in the dataset is complete, although the imputations are just placeholders. Next, the columns in the dataset are ranked according to the proportion of missing data, starting with the column containing the fewest missing values. For each column, the algorithm builds a predictive model using random forests, where the observed (non-missing) values in that column serve as the outcome variable, and the values from other columns for the same rows are used as predictors. Once predictions are generated, the initial guesses are replaced by these predictions. After cycling through all variables with missing values (one full iteration), the algorithm repeats the process several times (Stekhoven & Bühlmann, 2012), with a default of 10 iterations. During each iteration, the imputed values from the previous step are updated using the predictions from the random forest models trained in the current iteration. As the iterations proceed, the imputations are refined, as the predictions are based on increasingly accurate imputed values for the other variables. The algorithm stops after a predefined number of iterations.

A faster and extended version of the algorithm is given by the missRanger package of Mayer (2021). It uses the run-time efficient *ranger* implementation of random forest and additionally extends missForest by offering the option of *predictive mean matching* (PMM; Mayer, 2021).

PMM enhances random forest imputation by ensuring that imputed values are more realistic. Instead of directly using predicted values from the random forest, PMM matches each missing entry to one of the *k* closest observed values, the so-called donors. The actual observed value from this match is then used for imputation, maintaining the variability of the original data. PMM is particularly useful when it is important to faithfully reflect the underlying data distribution and impute values that are plausible within the observed range (Mayer, 2021). The number of donors is denoted by *k*. The default value is *k* = 5, which we also use in our study.

### MI through extreme gradient boosting (XGBoost)

Extreme gradient boosting (XGBoost) is a machine learning algorithm that belongs to the family of gradient boosting methods (Chen & Guestrin, 2016). Instead of simply averaging multiple decision trees as in random forests, XGBoost uses gradient boosting to combine multiple regression trees. It also employs regularization and shrinkage techniques. Typically, the depth of the regression trees used in sequential boosting are not very deep. One advantage of XGBoost over random forest is that XGBoost selects only one of a set of highly correlated features, because in sequential boosting, features are added to the model incrementally. If a feature is selected early, other highly correlated features can only improve the prediction if they provide new information.

XGBoost uses gradient descent optimization techniques to iteratively minimize the loss function, making it highly efficient and effective in finding the optimal solution. L1 and L2 regularization prevent overfitting and improve generalization. Tree pruning removes unnecessary branches and reduces model complexity, further enhancing XGBoost’s predictive performance.

Lastly, XGBoost can take advantage of parallel processing capabilities, making it suitable for large datasets and reducing training time. Overall, XGBoost is known for its ability to handle complex datasets and provide accurate predictions. Its popularity is evident in various domains, including Kaggle competitions and real-world applications (Chen & Guestrin, 2016).

The mixgb R package uses XGBoost to implement missing value imputation in a scalable and efficient manner (Deng & Lumley, 2024). It addresses the challenge of missing data in large datasets with complex structures with an iterative or non-iterative strategy parallel to chained random forest imputation. Initial values for imputation are filled with random values drawn from the observed data to initialize the imputation process (Suh & Song, 2023). mixgb imputes variables with fewer missing values first. This strategic approach aims to prioritize variables with more available information early in the imputation process. mixgb provides a versatile approach to missing data imputation, leveraging XGBoost, subsampling, and PMM to enhance imputation accuracy, especially for continuous data. The default imputation in mixgb is non-iterative but the package allows users to set the number of iterations for imputation (Deng & Lumley, 2024). For this simulation we used five iterations to be in line with the other imputation methods.

### Adapting tree-based imputation methods with cluster membership dummy variable

By default, missRanger and mixgb do not account for the multilevel structure of hierarchical data. To address this, we adapted these tree-based methods by incorporating dummy variables for cluster membership (denoted.dummies in the following), following the approach of Lüdtke et al. (2017). For *J* clusters, *J* dummy variables were added, with the *j*th variable equal to 1 if the observation belongs to cluster *j* and 0 otherwise. Unlike regression-based methods, tree-based methods do not require a reference group, as their decision trees can model cluster-specific effects without explicit parameterization (Breiman, 2001; Chen & Guestrin, 2016). Cluster-specific predictions in missRanger and mixgb, such as those using level 2 dummy variables or cluster means, are appropriate in our simulation, which uses the entire dataset to approximate the complete data distribution. However, in analyses with a training/test split, such predictions risk data leakage if test set information (e.g., cluster-level predictors) is used in the imputation of training data, and strict separation of training and test sets is required. Out-of-sample predictions for previously unobserved clusters are not naturally supported by this approach, as no cluster-specific effects can be learned or extrapolated beyond the observed clusters.

#### Multilevel structure

The dummy variables enable missRanger and mixgb to capture between-cluster effects by modeling cluster-specific differences in predictor values (in our case means of level 2 variables). Within-cluster effects are addressed by including level 1 variables as predictors in the imputation formula, which account for individual-level relationships. The flexibility of random forests (missRanger) and gradient boosting (mixgb) allows these methods, in principle, to capture nonlinear effects and interactions between predictors and dummy variables, potentially approximating variations akin to random slopes in more complex settings. However, unlike MICE, which explicitly models random intercepts and slopes using 2l.norm and a tailored predictor matrix (Grund et al., 2018a), tree-based methods do not directly estimate random effects but rely on algorithmic approximations, which differ conceptually from parametric multilevel specifications. The multilevel structures considered in this study are limited by the data-generating process and the analysis model. In particular, level 1 predictors are not decomposed into within- and between-cluster components, such that contextual effects of level 1 variables are not included. Cross-level interactions and other more complex multilevel dependency structures are likewise not part of the simulation design.

#### Level 2 variables

Tree-based methods, such as missRanger and mixgb, and MICE handle missing values in multilevel data differently, particularly for level 2 variables. For missRanger and mixgb, level 2 variables are imputed at the individual level using tree-based prediction models that include level 1 variables and cluster membership indicators as predictors (Deng, 2023; Mayer, 2021). As a result, cluster-level information enters the imputation process indirectly through the predictor set rather than through an explicit multilevel imputation model. In contrast, MICE’s 2lonly.pmm method imputes level 2 variables directly at the cluster level, generating a single imputed value per cluster and thereby explicitly enforcing within-cluster consistency and the hierarchical structure of the data (Grund et al., 2018a).

If regression trees have sufficient depth, the flexibility gained by the dummy variable approach can, in principle, exceed that of procedures that include only random intercepts, since some interactions with dummy variables are possible, although not all kinds of interactions (Wright et al., 2016). Potentially, more dummy variables are added than there are variables originally, so the dummy variable procedure relies on imputation methods that have some form of regularization. Tree-based ensemble methods like random forest and XGBoost do have this property by design (Breiman, 2001; Chen & Guestrin, 2016).

The tree-based methods missRanger and mixgb incorporate stochastic elements to introduce variability across imputations and thereby approximate aspects of “proper” imputations in the sense of Rubin (1996). Specifically, missRanger relies on random forests with bootstrapping and random feature selection, while mixgb introduces randomness through stochastic gradient boosting and subsampling. These mechanisms induce variation between imputations and partially propagate uncertainty related to both the missing values and the fitted imputation models. However, unlike fully parametric or Bayesian MI approaches, the tree-based procedures considered here do not generate imputations via explicit draws from a posterior predictive distribution. As a result, uncertainty propagation is achieved heuristically rather than through a formally specified probabilistic model.

## Simulation study design

### Simulated data

For the simulation setup, four general factors are varied, resulting in a total of 24 simulation designs. The varying factors are the *number of clusters*, *data generation model*, *missing rate*, and *missing mechanism*. We consider two numbers of clusters: 25 and 50. These choices reflect typical applied scenarios, with 25 clusters representing a small-sample setting (<30 clusters) and 50 clusters approximating asymptotic conditions (Cameron & Miller, 2015). This distinction matters practically, as inference methods perform differently depending on whether the number of clusters is small or large. This results in two cluster sizes of 40 and 20 for a balanced design with a sample size of *N* = 1,000. This variation in the number of clusters allows us to examine the effect of cluster size on the performance of the imputation methods. This fixed sample size limits the design to two combinations (25 clusters × 40 units, 50 clusters × 20 units), rather than a fully crossed design. This choice balances computational feasibility with scenarios common in applied multilevel research. The data generation models included a random intercept model and a random intercept and slope model, capturing varying degrees of multilevel complexity. Missingness rates were set to 10%, 30%, and 50% to cover a range of practical scenarios, and missingness mechanisms were restricted to missing completely at random (MCAR) and missing at random (MAR), following standard practices (Little & Rubin, 2002). We evaluated each condition using bias, standard deviation, type I error rates, and power.

### Data generation

To assess the impact of these factors, we simulate data in a controlled manner.

Specifically, we generate data by using the fungible package in R (Waller, 2022). The monte function generates clustered data with predefined characteristics. We generate 12 continuous predictor variables: six level 1 variables (*X*_*ij*1_ to *X*_*ij*6_) and six level 2 variables (*L*_*ij*1_ to *L*_*ij*6_). The level 1 variables were drawn from a multivariate normal distribution, with correlations and intraclass correlation coefficients (ICCs) specified by *η*^2^ ∼ Uniform(0*,* 0*.*85) to reflect varying clustering strength typical in social science research (Snijders & Bosker, 2012). Level 2 variables were computed as the cluster means of the corresponding variables generated by fungible::monte. This process ensures that level 2 variables are correlated with level 1 variables, facilitating imputation by capturing hierarchical relationships (van Buuren, 2011).

Finally, two data generation models are considered for the outcome variable: *random intercept *and *random intercept and random slope* (hereafter denoted as *random slope* model). The data-generating process assumes a linear multilevel model, aligning with the assumptions of the linear mixed model used for analysis and the MICE imputation method (2l.norm), which may favor MICE over other methods like missRanger or mixgb that do not explicitly assume linearity (van Buuren, 2018). Based on the model, the output variable is entered as follows.

- Random intercept model:
  $${Y}_{ij}={\beta }_{0}+{\beta }_{1}{X}_{ij1}+{\beta }_{2}{X}_{ij2}+{\beta }_{3}{X}_{ij3}+{\beta }_{4}{X}_{ij4}+{\beta }_{5}{X}_{ij5}+{\beta }_{6}{X}_{ij6}+{\beta }_{7}{L}_{ij1}+{\beta }_{8}{L}_{ij2}+{\beta }_{9}{L}_{ij3}+{\beta }_{10}{L}_{ij4}+{\beta }_{11}{L}_{ij5}+{\beta }_{12}{L}_{ij6}+{\delta }_{0j}+{\epsilon }_{ij},$$

where ${\delta }_{0j}\sim N\left(\mathrm{0,10}\right), {\epsilon }_{ij}\sim \left(\mathrm{0,1}\right)$, and fixed coefficients are *β* = (0*.*3*,* 0*.*5*,* 1*,* 1*.*5*,* 0*,* 0*,* 0*,* 0*.*5*,* 1*,* 1*.*5*,* 0*,* 0*,* 0).

- Random intercept and slope model:where *δ*_0*j*_*, δ*_1*j*_*, δ*_2*j*_ ∼ N(0*,* 1), *ϵ*_*ij*_ ∼ N(0*,* 10), and the same fixed coefficients apply.
  $${Y}_{ij}={\beta }_{0}+{\beta }_{1}{X}_{ij1}+{\beta }_{2}{X}_{ij2}+{\beta }_{3}{X}_{ij3}+{\beta }_{4}{X}_{ij4}+{\beta }_{5}{X}_{ij5}+{\beta }_{6}{X}_{ij6}+{\beta }_{7}{L}_{ij1}+{\beta }_{8}{L}_{ij2}+{\beta }_{9}{L}_{ij3}+{\beta }_{10}{L}_{ij4}+{\beta }_{11}{L}_{ij5}+{\beta }_{12}{L}_{ij6}+{\delta }_{0j}+{\delta }_{0j}{X}_{ij1}+{\delta }_{2j}{X}_{ij2}+{\epsilon }_{ij},$$

In the case of the random slope model, *δ*_0*j*_*, δ*_1*j*_*, δ*_2*j*_*, *and *ϵ*_*ij*_ are randomly generated. The six noise features *X*_*ij*4_*, X*_*ij*5_*, X*_*ij*6_*, L*_*ij*4_*, L*_*ij*5_*,* and *L*_*ij*6_ are uncorrelated with the output variable *Y*_*ij*_. They are included to enable evaluation of the type I error and to assess the ability of the imputation methods to distinguish between important and unimportant variables. The variance of the error terms was set to 10 to achieve a realistic marginal *R*^2^. We have a marginal *R*^2^ of 0.484 for the random intercept model and 0.442 for the random intercept and random slope model. The fixed coefficients (*β*_1_ = 0*.*5, *β*_2_ = 1, *β*_3_ = 1*.*5, *β*_7_ = 0*.*5, *β*_8_ = 1, *β*_9_ = 1*.*5, others zero) were chosen to reflect small to moderate effect sizes typical in social science multilevel studies according to Maas and Hox (2005). Our values, with standardized coefficients ranging from approximately 0.10 to 0.33, provide a realistic testbed for evaluating the ability of imputation methods to recover subtle effects in hierarchical data, where clustering often reduces effect magnitudes. The variance components for random effects were set to 1 to standardize their contributions and simplify comparisons across conditions, though this may not reflect the heterogeneity of real-world data (Snijders & Bosker, 2012.

Missing values were introduced at both level 1 and level 2. After generating the data, missing values were introduced in the level 2 predictors (*L*_*ij*1_*,..., L*_*ij*6_) at rates under MCAR and MAR mechanisms at the cluster level. These missing values were imputed using five methods: MICE, missRanger (with and without dummies) and mixgb (with and without dummies). The imputed datasets were analyzed using the linear mixed model with random intercepts, matching the data-generating model. We evaluated the performance of the imputation methods by computing the type I error rate (the proportion of significant results for predictors with true coefficients *β*_4_ = *β*_5_ = *β*_6_ = *β*_10_ = *β*_11_ = *β*_12_ = 0 at *α* = 0*.*05), power, coefficient bias and standard deviation of the parameter estimation. These metrics assess the impact of imputation on the subsequent multilevel analysis, rather than directly measuring the accuracy of imputed values. This choice was made because practitioners are usually more interested in inference multilevel analysis.

### Missingness mechanism

Missingness in the generated data is induced at three levels from moderately low to relatively high (10%, 30%, and 50%). This range of missingness levels reflects real-world scenarios. Two missingness mechanisms (MAR, MCAR) are considered. The introduction of missing data is based on the selected missingness mechanism and the specified missing rate. For MCAR, a simple function is implemented in R that randomly sets each data point to NA with probability equal to the missingness level for each variable. For MAR, missingness in level 1 variables (*X*_*ij*1_*,..., X*_*ij*6_, *Y*_*ij*_) was implemented using a custom function based on Thurow, Dumpert, Ramosaj, and Pauly (2021a, b). The algorithm operates as follows: Starting with one variable, it generates missing values under MCAR with the overall missing rate. The values of the selected variable were binned into 25 equal-width intervals based on the variable’s range, and missingness in other level 1 variables (*X*_*ij*2_*,..., X*_*ij*6_, *Y*_*ij*_) was assigned with probabilities proportional to the frequency of non-missing values in each interval, adjusted by random weights drawn from 𝘚(0*,* 1). This ensures that missingness depends on the observed values of the selected variable, satisfying the MAR condition. For 50% missingness, the miss rate of the reference column was set to 30% in order to avoid intervals only containing missing values. The missingness of the level 2 variables was induced via MCAR in both scenarios. The resulting simulation has 2 × 2 × 2 × 3 = 24 conditions. Each combination of the above factors is replicated 1,000 times.

### Treatment of missing data and choice of hyperparameters

Missing data were imputed using three methods: MICE, missRanger, and mixgb. For MICE, a multilevel approach was employed using the mice package (van Buuren & Groothuis-Oudshoorn, 2011), applying 2l.norm for level 1 variables (*X*_*ij*1_*,..., X*_*ij*6_, *Y*_*ij*_) to model random intercepts by cluster. For the level 2 variables (*L*_*ij*1_*,..., L*_*ij*6_) we used 2lonly.pmm, i.e., predictive mean matching at the cluster level with other level 2 variables as predictors. This approach accounts for clustering but does not include random slopes. The exclusion of random slopes was intentional, as it avoids further favoring MICE, which was already advantaged by the simulation’s linear mixed model design compared to the tree-based methods. After imputation, we fitted the analysis model to each completed dataset and combined the results using Rubin’s rules (Little & Rubin, 2002).

We went with the default choices of hyperparameters if not stated elsewise (Deng, 2023; Mayer, 2021; van Buuren & Groothuis-Oudshoorn, 2011). For mice, the number of multiple imputations was thus set to *m*=5, and the number of iterations was set to maxit=10. Moreover, PMM was used with five donors. For the random forest and the ranger package, there are more hyperparameters that the user has to choose. We used fully grown trees corresponding to max.depth=NULL in the ranger function. For mtry and sample.fraction we also stayed at the default value, which was the rounded-down root of the number of features and 0.632 times the sample size, respectively. Since the random forest is computationally expensive, we reduce the number of trees to speed up the simulation and set num.trees=300 instead of the default value of 500, because the expected performance loss is negligible (Probst & Boulesteix, 2018). As a splitting rule for ranger, we used the traditional variance minimization from the CART (Classification and Regression Trees) algorithm (Breiman, 2001; Breiman et al., 1984). An alternative conditional inference-based splitting criterion was introduced by Hothorn et al. (2006), which can reduce variable selection bias toward variables with many cut points (Strobl et al., 2007). This bias is inherent in traditional CART-based splitting rules and can also affect permutation importance measures. Such bias could, in principle, also affect downstream analyses. However, in our imputation context, the primary objective is to obtain plausible values that enable valid subsequent inferential analysis rather than estimation of variable importance. To our knowledge, no adaptation of the conditional inference-based splitting criterion for use in imputation currently exists. Moreover, as the standard random forest as implemented in ranger has great predictive performance (Grinsztajn et al., 2022), we expect that these properties are beneficial for imputation as well. For the predictive mean matching step, the number of candidates to sample from is set to 5. For the mixgb imputation method, we also use mostly default hyperparameter settings with two exceptions: The number of multiple imputations is set to *m* = 5 for better comparability with the other imputation approaches. For mixgb we use the default of histogram-based splitting (Deng, 2023). To balance computational efficiency with sufficient convergence, we limit the number of iterations for the imputation process to maxit = 5. These values reflect a compromise between computational cost and the stability of imputations needed for robust estimates. Prior to the main simulation, we conducted a tuning study to assess whether hyperparameter optimization could meaningfully improve the performance of mixgb. We performed a grid search across key parameters (including num_boost_round, max_depth, eta, and subsample) in a reduced simulation setting with 100 replications, optimizing root mean square error (RMSE). For each condition, we compared the statistical power obtained with mixgb’s default parameters to the power obtained with the best-performing (according to RMSE) hyperparameter configuration identified through a structured tuning grid search.

However, the results showed that optimized settings improved power by at most 0.02 on average, and the mean absolute change in power across all variables and scenarios was approximately 0.01 (see Table 1 and Table 2 in the Appendix). Given the negligible benefit relative to the substantial additional computational cost, we decided to use the default hyperparameter settings for mixgb in the main simulation study.

## Simulation study results

In this simulation study, various imputation methods were evaluated and compared to address missing data in multilevel designs. The primary objective was to assess the performance of these methods with respect to subsequent inference across different settings. To this end, the methods were evaluated using rejection rates and coefficient bias. Rejection rates assess type I error control (in the case of a true null hypothesis) and statistical power (in the case of a false null hypothesis), while coefficient bias provides insight into the precision and reliability of the estimated parameters. Comparing the standard deviation of the parameter estimation in the imputed data with that in the complete data quantifies the uncertainty of the estimation caused by imputation. Evaluating different settings helps to assess the overall robustness of the imputation methods.

Since the overall conclusion from the results of the random intercept model aligns with that of the random slope model, we focus on discussing the former. The results for the random slope model are provided in the supplement.

### Rejection rates

The Monte Carlo standard error (MCSE) for power and type I error rates was calculated following Morris et al. (2019) as

$$\sqrt{\frac{p\left(1-p\right) }{N}}$$

where *p* is the fraction of significant results and *N* = 1*,* 000 is the number of replications, ranging from 0.0010 to 0.0158.

#### Type I error

**Fig. 1:**
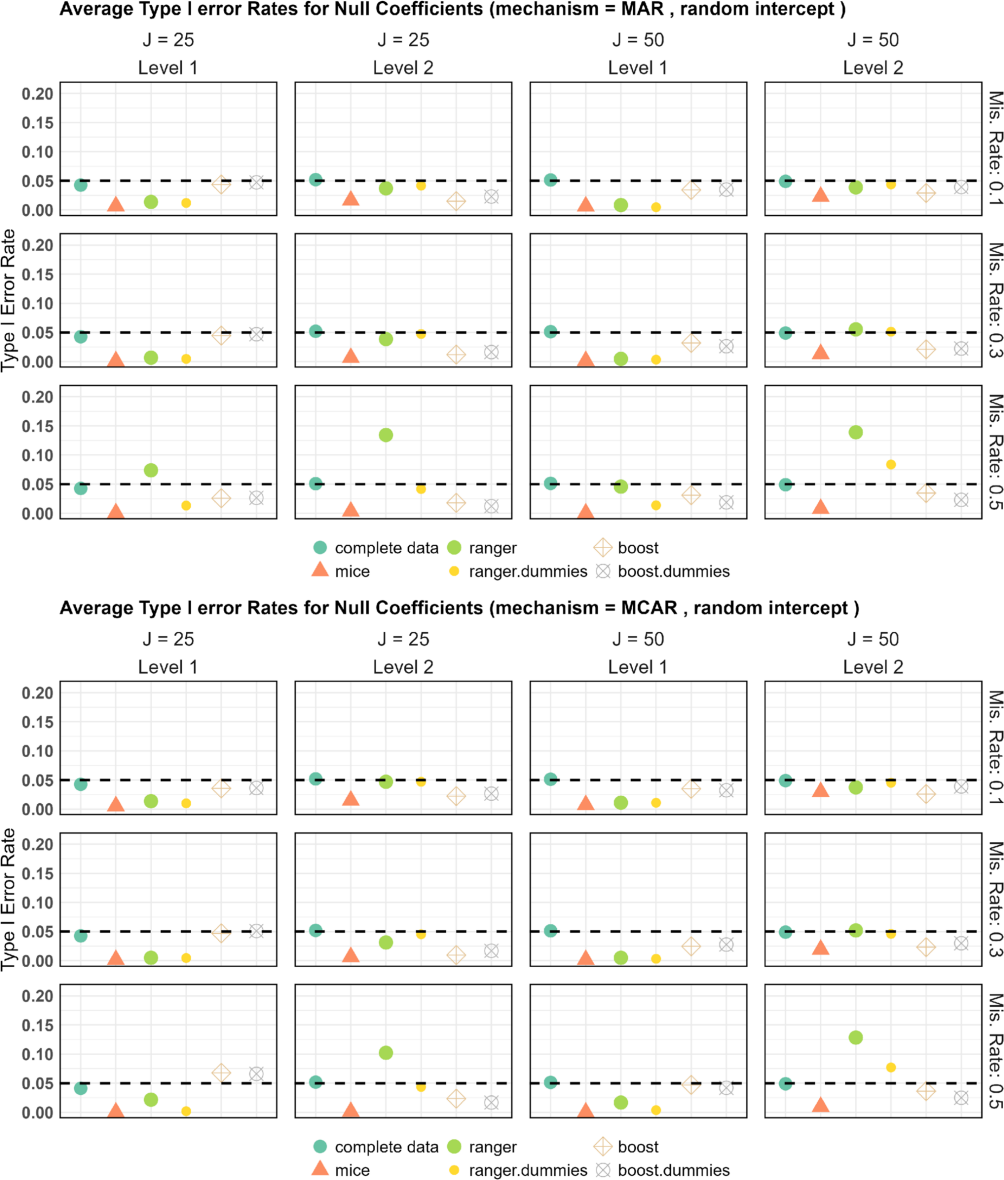
Estimated type I errors for random intercept designs

Figure 1 shows the average rejection rates of *H*_0_ across replications for random intercept models, categorized by the missingness mechanism. Here, *H*_0_ states that the variable (for level 1) or the cluster variable (for level 2) has no effect on the dependent variable. Only coefficients that are truly zero are included in the figure, thus reflecting the estimated type I errors. As seen in the subfigures of Fig. 1, missranger without predictive mean matching (displayed in the figures as ranger) exceeds the significance level (with and without dummies) for every coefficient across all combinations of missingness mechanism, missing rate, and cluster size. In some cases, the rejection rates are larger than 10%, despite the significance level being set to 5%, highlighting the importance of PMM. Consequently, we exclude random forests without PMM from the subsequent power analysis. For a cluster size of 25 and 50% missingness, the type I error with missranger and predictive mean matching (displayed as ranger5) is larger than desired for level 2 features, though the test maintains the significance level when using dummies. This suggests that dummies can be crucial. However, for a cluster size of 50 and 50% missingness, all random forest approaches result in estimated type I errors exceeding 0.05 for the level 2 features. This holds for both MCAR and MAR. Notably, for the level 1 variables without an effect, the null hypothesis (*H*_0_) was rejected in almost none of the cases for MICE and missranger with PMM, i.e., the type I error rate is close to zero, indicating that these methods are overly conservative.

As shown in Fig. 1, among the different imputation methods evaluated, only MICE, ranger.dummies with PMM, and mixgb (both with and without dummies) consistently achieved a type I error rate below 5%, while MICE was the only method that was always below 5%.

#### Power

**Fig. 2:**
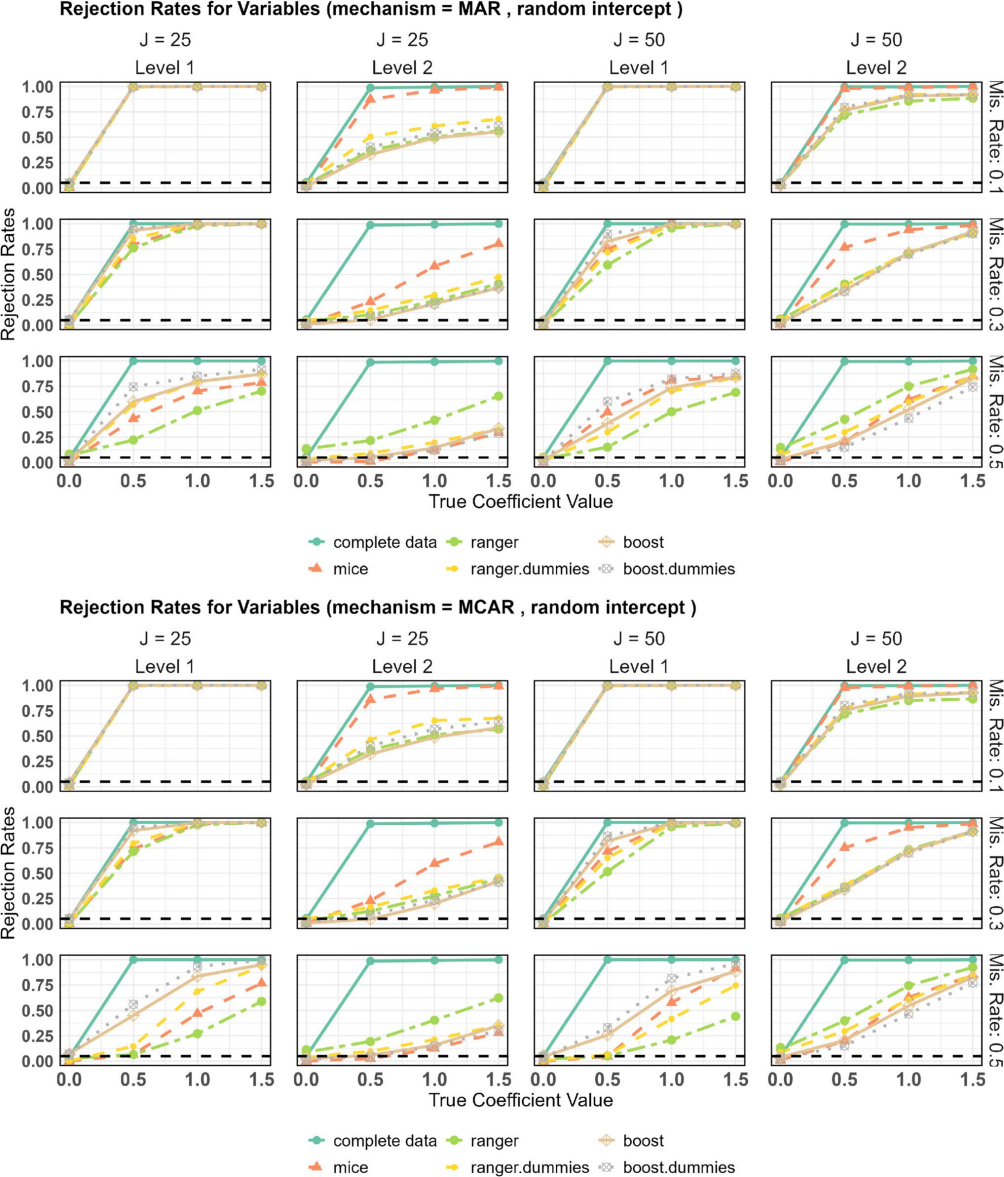
Power for random intercept designs

Figure 2 displays the rejection rates for the nonzero coefficients, i.e., the estimated power. While the plots for MAR and MCAR are very similar, the missingness rate clearly plays an important role: For 10% missingness, the level 1 effects are almost always detected with only minor differences between the imputation methods. However, for the level 2 variables, MICE outperformed the other methods, with a power more than 0.3 higher than the others for a cluster size of 25. For 30% missingness, MICE remains the benchmark for detecting effects on level 2, but for the level 1 variables, differences between the imputation methods start to occur: The power function at 0.5 for mixgb and mixgb with dummies is better than the power of MICE, where the variants with dummies seem to have a slight advantage. This holds independently of the cluster size.

This remains consistent across all cluster sizes. The variants of missranger performed worse than MICE and the mixgb methods. At 50% missingness, the advantage of mixgb with dummies for level 1 variables becomes more pronounced. For each cluster size and missingness mechanism, mixgb with dummies sets the benchmark. Particularly for MCAR, mixgb with dummies significantly outperforms MICE. For level 2 variables, mixgb with dummies performs similarly to MICE at a cluster size of 25, whereas at a cluster size of 50, MICE has a slight advantage. This pattern is consistent for both missingness mechanisms. Therefore, at 50% missingness, mixgb with dummies emerges as the preferred method, even though MICE benefits from knowing the linear structure of the model equation.

### Coefficient bias

**Fig. 3:**
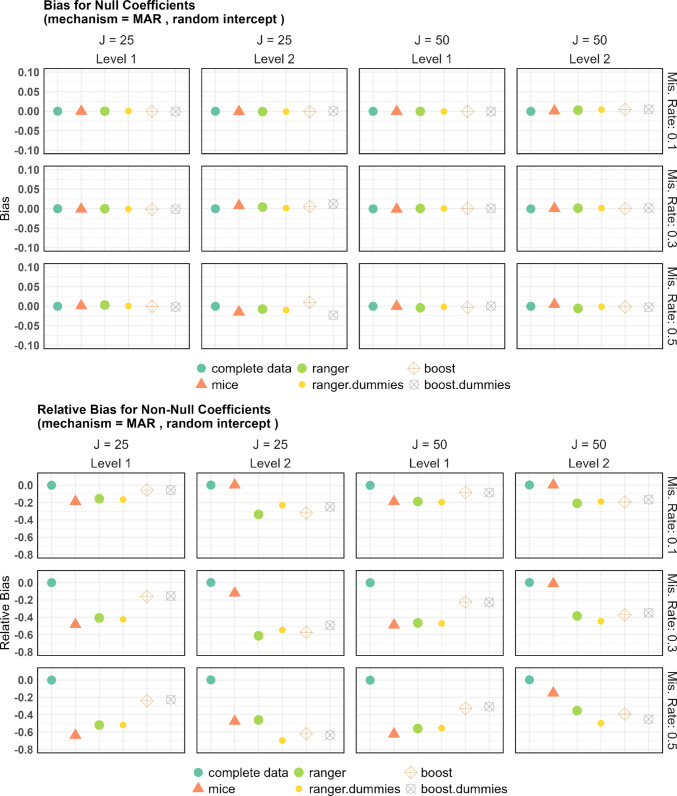
Bias for the true zero coefficients and relative bias for the nonzero coefficients

For the bias of zero coefficients, the MCSE was estimated as $\frac{\mathrm{SD}}{{\surd }_{N}}$ using the standard deviation (SD) from simulation data, with values between 0.0044 and 0.0601. The MCSE for the relative bias of nonzero coefficients was computed similarly, ranging from 0.0407 to 0.2353, where all MSCE errors of more than 0.2 occurred, when missingness was 50%. Figures 3 shows the coefficient bias for each method under random intercept models with MAR. For the true zero coefficients at both level 1 and level 2, we observe that the bias values are consistently close to zero. Even with increasing missingness or decreasing cluster size, no significant deviations from zero are found. A more interesting observation is the relative bias (adjusted for effect size) for the nonzero coefficients. The relative bias is used to normalizes the bias by the true parameter value, making it easier to interpret and compare across different coefficient sizes. It appears that all imputation methods induce negative bias. These findings regarding relative bias align closely with the rejection rates observed earlier. mixgb, both with and without dummies, exhibits relatively high relative bias for the level 2 variables. In contrast, MICE produces the lowest bias among the tested imputation methods for level 2 variables, regardless of the missingness rate. On the other hand, for level 1 variables, MICE shows larger bias than the boosting methods, and this difference increases with increasing missing rate. The random forest methods also show relatively large bias for both level 1 and level 2 variables.

### Standard deviation

**Fig. 4:**
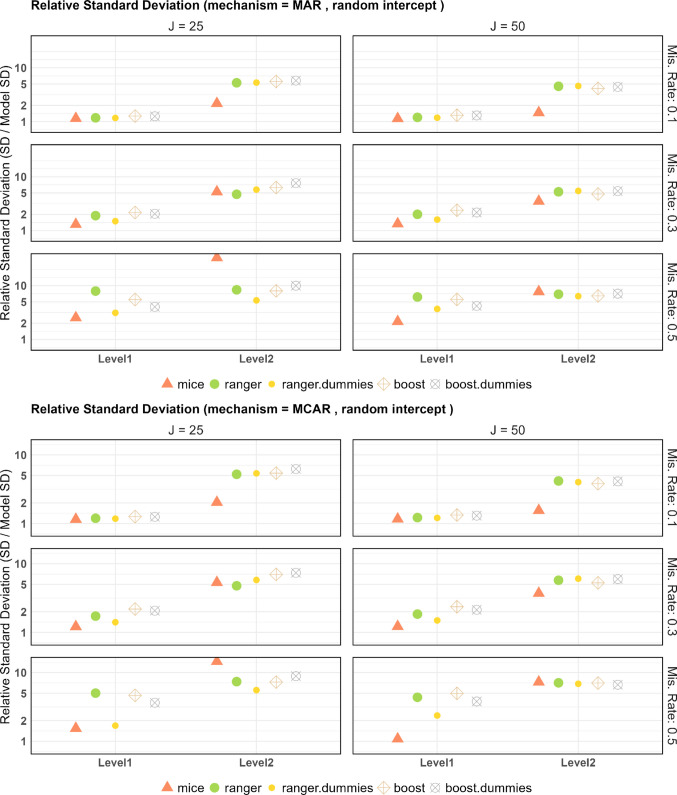
Ratio of standard deviation (imputed data vs. complete data) for the random intercept model

For the standard deviation of nonzero coefficients, the MCSE was determined as $SD/{}^{\surd }2N$ (Morris et al., 2019), based on the standard deviation, ranging from 0.0023 to 0.22236 (the larger values only occurred occasionally for 50% missingness). Figure 4 shows the ratio of the standard deviation of parameter estimates from the imputed data to the standard deviation of parameter estimates from the complete dataset. The imputation increases the standard deviation for (almost) every combination of parameter, cluster size, missing mechanism, miss rate, and imputation method.

For the level 1 features, the increase in the standard deviation seems rather small at a 10% miss rate. At a miss rate of 30%, the imputation with mixgb (with or without boosting) doubles the standard deviation of the parameter estimation. In this case, MICE performs best at preserving the variance of the complete dataset. The difference becomes even more drastic at a 50% miss rate: the standard deviation for MICE stays robust for the true-zero features, while the other imputation methods multiply the standard deviation of parameter estimation. For the nonzero features, MICE remains best at preserving the standard deviation, but the difference relative to the other methods is smaller. At a 50% miss rate, MICE’s standard deviation is not as robust for the nonzero features as for the true-zero features, but is still better than the tree-based alternatives. Thus, the power advantage of mixgb for level 1 features at the larger miss rates comes from a smaller bias (Fig. 3).

For the level 2 features, the inflation of standard deviation is much higher than for the level 1 features, especially for larger miss rates. For a cluster size of 25 and 50% missingness, the standard deviation on the MICE imputed data is more than 10 times that on the full dataset. This also explains why mixgb has more power on level 2 (Fig. 1) despite having larger negative bias (Fig. 3). In this scenario, ranger is the best at preserving the original standard deviation. The standard deviation of MICE remains worse than the other imputation methods for the cluster size of 50. At a miss rate of 10%, MICE preservation is superior.

**Fig. 5:**
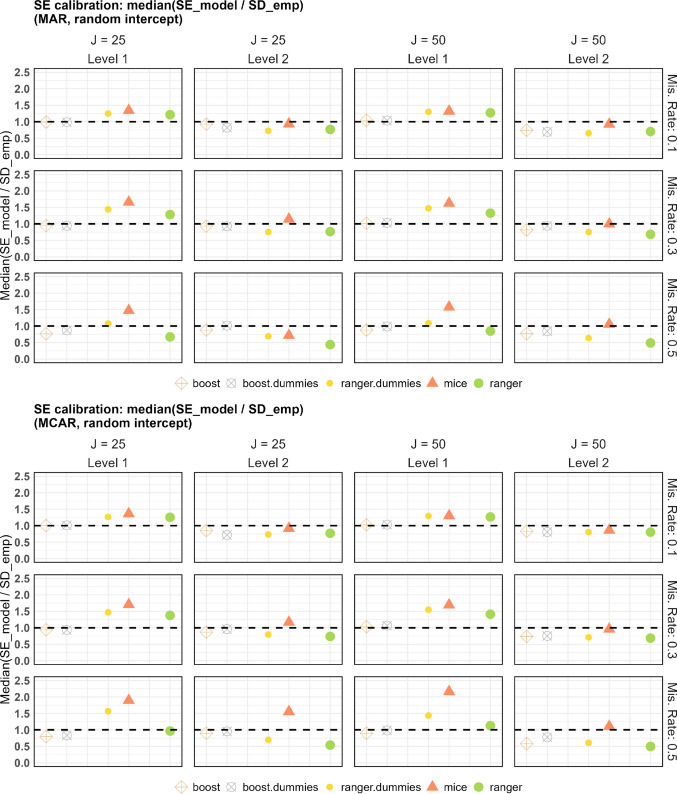
Calibration of model-based standard errors for the random intercept model. Ratios of average model-based standard errors to empirical standard deviations across replications are shown

In addition to rejection rates and empirical standard deviations, we evaluated the calibration of model-based standard errors by examining the ratio of the average reported standard errors to the empirical standard deviation of the parameter estimates across replications (Morris et al., 2019). Ratios close to 1 indicate well-calibrated uncertainty estimates, whereas deviations reflect under- or overestimation of uncertainty. Figure 5 shows that boost and boost.dummies yield ratios close to 1 in the random intercept model. Results for the random intercept and random slope model are similar and are shown in Fig. A5 in the Appendix. For mice, model-based standard errors tend to be larger than the empirical standard deviations for level 1 parameters, particularly for higher missing rates and *J* = 50. In contrast, the variants of ranger (with and without dummy variables) show substantial deviations from 1, indicating both under- and overestimation of standard errors across simulation conditions also depending on the use of PMM.

## Discussion

This paper critically evaluates the performance of novel tree-based imputation methods for handling missing data in hierarchical data structures, focusing on random forest (missranger)- and XGBoost (mixgb)-based approaches with and without dummy modeling.

Through a comprehensive simulation study, we contrast these novel techniques with the state-of-the art MICE approach, focusing in particular on bias and inference. The evaluation considers level 1 and level 2 variables across two different data generation processes (random intercept and random slope models), three missingness rates (10%, 30%, and 50%), and two missingness mechanisms (MCAR and MAR). Our results indicate that MICE is characterized by consistent accuracy in rejection rates when using the 2l.norm method for level 1 variables and 2lonly.pmm for level 2 variables. This consistency underscores MICE’s robustness to hierarchical data and closely approximates true rejection rates (i.e., type I error and power). Our results are consistent with the findings in Grund et al. (2018a), demonstrating the practical usefulness of MICE when dealing with missingness in multilevel structures, especially in the case of missingness at level 2. Similar to Enders et al. (2016), we also found that MICE was superior not only for random slope but for random intercept models, provided that the imputation model was specified according to the data generation process and the missing rate was low (10%).

For level 1 coefficients, boosting (mixgb) with dummies consistently outperforms other methods, including MICE, across all missingness mechanisms when the missingness rate is 30% or 50%. At 30% missingness, however, MICE has more power for level 2 variables, making the choice of imputation method dependent on the practitioner’s priorities and the relative importance of level 1 versus level 2 variables in the specific application. At 50%, mixgb (with and without dummies) proved to be the preferable alternative in comparison to MICE regardless of missing mechanism or cluster size. The analysis of (relative) coefficient bias corroborates the findings from the rejection rates, revealing larger relative bias for MICE in level 1 variables and greater relative bias for the boosting methods in level 2 variables. The missranger methods, on the other hand, fell slightly behind the boosting methods and MICE for almost all settings and cannot be recommended as an imputation method in our multilevel settings. In particular, missranger without PMM showed substantially increased type I errors.

While the rejection rate differences between imputation methods vary slightly across the random intercept and random slope models, the overall trends remain consistent regardless of the data-generating process. The results do not necessarily indicate that tree-based methods with dummies (also including dummies for the level 2 cluster) outperform their standard counterpart without dummies. For example, under MCAR and MAR, standard mixgb showed slightly higher power than mixgb with dummies for level 2 coefficients when the missingness rate was 50% in both random intercept and random slope. Meanwhile, adjusted mixgb sometimes exhibited higher power for level 1 coefficients. In some situations (e.g., 50% missingness and cluster size 25), the introduction of dummies helped to decrease the type I error to the desired level. But for most scenarios the introduction of dummies did not alter the results much.

## Limitations, strengths, and outlook

Our simulation study provides valuable insights into imputation methods for hierarchical data, but its generalizability is limited by the design of the data-generating process (DGP). The DGP assumes a linear multilevel model, aligning closely with the assumptions of the linear mixed model used for analysis and the MICE imputation method (2l.norm) (van Buuren, 2018). This alignment likely favors MICE, particularly at low missingness rates (10%), as 2l.norm explicitly models linear and hierarchical relationships, unlike missRanger or mixgb, which are more flexible but do not assume linearity. Consequently, our results may not generalize to settings with nonlinear relationships (e.g., jumps or polynomial terms) or higher-order interaction effects, where tree-based methods could leverage their ability to capture complex patterns. The use of a low number of variables in our simulation further limits generalizability to complex, variable-rich datasets typical in real-world social science applications. The fact that mixgb was still able to outperform MICE for increasing missing rates underlines the potential of tree-based imputation methods for multilevel data.

Future research investigating the impact of nonlinear variable effects and a larger number of variables on both rejection rate and bias could provide a more complete understanding of the methods’ performance in more complex data settings. In particular, simulating data beyond a linear mixed model (e.g., with jumps or non-monotonic effects) could be insightful, such as using a semi- or nonparametric data-generating model that remains plausible enough for a linear mixed model analysis. In addition, the tree-based methods missRanger and mixgb rely on traditional splitting algorithms (variance minimization and histogram-based, respectively), which may introduce bias in variable selection for certain tasks. Alternative approaches, such as conditional inference trees (Hothorn et al., 2006), could reduce such biases but are not yet adapted for imputation, presenting a promising direction for future research.

For empirical researchers, the alignment between DGP, imputation method, and analysis model is critical for imputation quality. When the underlying DGP is likely linear and hierarchical, as is common in the social sciences (e.g., education, psychology) (Maas & Hox, 2005), MICE with 2l.norm or 2lonly.pmm is recommended due to its robustness and ability to preserve multilevel structures. For larger missing rates, mixgb can be a viable alternative. For other DGPs, further research is needed.

In addition, the multilevel structures considered in this study are limited. Although random intercepts and random slopes are included, more complex multilevel features such as contextual effects of level 1 predictors, cross-level interactions, or alternative centering strategies are not part of the DGP. Addressing these more complex multilevel structures would require substantially different simulation designs and is therefore left for future research. A further limitation of the present simulation study concerns the assessment of inferential uncertainty. While we evaluated rejection rates, empirical standard deviations, and the calibration of model-based standard errors, we did not directly assess confidence interval coverage.

The strength of the current study, however, lies in its novelty. We introduce and evaluate the combination of the latest tree-based imputation methods and a simple multilevel adjustment via dummy variables against established techniques such as MICE. This approach provides new insights into the evolving landscape of data imputation methods, especially in the context of hierarchical data structures.

Another interesting area to explore would be to improve the performance of tree-based methods. This could involve experimenting with multivariate tree-based methods (Schmid et al., 2023; Sega & Xiao, 2011). Tree-based methods with random effects have been developed (Bergonzoli et al., 2024; Buczak, 2024; Fokkema et al., 2020; Fokkema et al., 2018; Hajjem et al., 2014, 2017; Sela & Simonoff, 2012), but still have to be adapted to missing data. Another possible approach could be to first average all data at level 2 and impute missing data at this aggregate level, and then merge only the level 2 variables with the individual level dataset. A further idea for future research is a comparison of (potentially improved) tree-based methods with model-based approaches of (Enders, 2023; Enders et al., 2020; Grund et al., 2021) or the evaluation of different regression models like an all-noise model or a model with more coefficients. Future research should also explore imputation methods for systematically missing level 1 features, which were not included in our simulation, focusing on MCAR and MAR mechanisms, to address more complex missing data scenarios (Jolani et al., 2015; Resche-Rigon & White, 2018).

In conclusion, our study underscores the continued effectiveness of MICE in dealing with hierarchical data, particularly in terms of rejection rates. However, the emerging tree-based methods, especially mixgb, show potential for larger missing rates, suggesting their usefulness as alternatives in certain contexts. This dual finding opens new avenues for future research and practical applications in data imputation, highlighting the dynamic nature of the field.

## Supplementary Information

Below is the link to the electronic supplementary material.

Supplementary file1 (PDF 2853 KB)

## Data Availability

The simulated datasets during the current study are available under https://drive.google.com/file/d/1BD1EhQtyBGYArkZpgOUR51eIQsHaqO5Z/view?usp=drive_link.

## Appendix

**Table 1:** Average power for default and tuned mixgb (model: random intercept) by missing rate

Mechanism	Miss rate	Variable	Mean default	Mean tuned	Difference
MAR	0.10	*L*21	0.33	0.33	0.00
MAR	0.10	*L*22	0.59	0.59	−0.00
MAR	0.10	*L*23	0.79	0.79	0.00
MAR	0.10	*X*_1_	0.52	0.52	0.00
MAR	0.10	*X*_2_	0.97	0.97	0.00
MAR	0.10	*X*_3_	1.00	1.00	0.00
MAR	0.30	*L*21	0.32	0.33	0.00
MAR	0.30	*L*22	0.60	0.60	−0.00
MAR	0.30	*L*23	0.78	0.79	0.02
MAR	0.30	*X*_1_	0.52	0.52	−0.00
MAR	0.30	*X*_2_	0.96	0.96	−0.00
MAR	0.30	*X*_3_	1.00	1.00	0.00
MAR	0.50	*L*21	0.32	0.33	0.00
MAR	0.50	*L*22	0.61	0.61	−0.01
MAR	0.50	*L*23	0.78	0.79	0.01
MAR	0.50	*X*_1_	0.50	0.52	0.01
MAR	0.50	*X*_2_	0.95	0.96	0.01
MAR	0.50	*X*_3_	1.00	1.00	0.00
MCAR	0.10	*L*21	0.30	0.30	0.00
MCAR	0.10	*L*22	0.56	0.56	−0.00
MCAR	0.10	*L*23	0.79	0.79	−0.00
MCAR	0.10	*X*_1_	0.48	0.49	0.01
MCAR	0.10	*X*_2_	0.96	0.96	0.00
MCAR	0.10	*X*_3_	1.00	1.00	0.00
MCAR	0.30	*L*21	0.27	0.28	0.01
MCAR	0.30	*L*22	0.56	0.55	−0.01
MCAR	0.30	*L*23	0.78	0.79	0.01
MCAR	0.30	*X*_1_	0.43	0.46	0.03
MCAR	0.30	*X*_2_	0.91	0.94	0.03
MCAR	0.30	*X*_3_	0.99	1.00	0.00
MCAR	0.50	*L*21	0.30	0.32	0.01
MCAR	0.50	*L*22	0.52	0.51	−0.01
MCAR	0.50	*L*23	0.74	0.75	0.01
MCAR	0.50	*X*_1_	0.46	0.49	0.03
MCAR	0.50	*X*_2_	0.81	0.83	0.02
MCAR	0.50	*X*_3_	0.98	0.99	0.01

**Table 2:** Average power for default and tuned mixgb (model: random intercept and random slope) by missing rate

Mechanism	Miss rate	Variable	Mean default	Mean tuned	Difference
MAR	0.10	*L*21	0.25	0.26	0.00
MAR	0.10	*L*22	0.51	0.52	0.00
MAR	0.10	*L*23	0.73	0.74	0.00
MAR	0.10	*X*_1_	0.49	0.49	−0.00
MAR	0.10	*X*_2_	0.92	0.92	0.01
MAR	0.10	*X*_3_	1.00	1.00	0.00
MAR	0.30	*L*21	0.26	0.24	−0.02
MAR	0.30	*L*22	0.52	0.52	0.01
MAR	0.30	*L*23	0.75	0.75	0.00
MAR	0.30	*X*_1_	0.49	0.50	0.01
MAR	0.30	*X*_2_	0.91	0.91	0.00
MAR	0.30	*X*_3_	0.99	0.99	0.00
MAR	0.50	*L*21	0.28	0.29	0.01
MAR	0.50	*L*22	0.52	0.52	0.00
MAR	0.50	*L*23	0.76	0.76	0.00
MAR	0.50	*X*_1_	0.48	0.49	0.01
MAR	0.50	*X*_2_	0.88	0.90	0.01
MAR	0.50	*X*_3_	0.99	0.99	0.00
MCAR	0.10	*L*21	0.23	0.23	0.00
MCAR	0.10	*L*22	0.56	0.55	−0.01
MCAR	0.10	*L*23	0.72	0.73	0.01
MCAR	0.10	*X*_1_	0.42	0.44	0.01
MCAR	0.10	*X*_2_	0.92	0.93	0.00
MCAR	0.10	*X*_3_	0.99	0.99	0.00
MCAR	0.30	*L*21	0.25	0.23	−0.01
MCAR	0.30	*L*22	0.56	0.56	−0.00
MCAR	0.30	*L*23	0.69	0.68	−0.01
MCAR	0.30	*X*_1_	0.41	0.47	0.06
MCAR	0.30	*X*_2_	0.84	0.88	0.04
MCAR	0.30	*X*_3_	0.99	0.99	0.00
MCAR	0.50	*L*21	0.24	0.24	−0.00
MCAR	0.50	*L*22	0.49	0.50	0.01
MCAR	0.50	*L*23	0.70	0.69	−0.01
MCAR	0.50	*X*_1_	0.42	0.43	0.01
MCAR	0.50	*X*_2_	0.75	0.80	0.05
MCAR	0.50	*X*_3_	0.97	0.98	0.01

## Footnotes

1 The default value for missRanger is 0, and the default value in MICE is 5. Not using PMM for missRanger showed inflated type I errors in another context (Ramosaj et al., 2020).

2 To choose a number of imputations, a mini-simulation on randomly selected design was run with 5 and 20 imputations with MICE and missRanger (standard and with dummies with three donors). The results suggested very little to no differences between the number of imputations in terms of coefficient estimation bias and *H*0 rejection rates, but imputing 20 times takes approximately four times longer than performing 5 imputations. Consequently, the whole simulation is run with 5 imputations for each method, making the simulation considerably faster.

3 2 l indicates that the data structure is multilevel; it does not mean the calculation happens at a higher level, but it considers that there are variables at another level.

## Code availability

The code used in this study is available under https://osf.io/8256p/overview.
