# Supplementary material for: Adapting tree-based multiple imputation methods for multilevel data? A simulation study
Source: Behav Res Methods. 2026 May 13;58(6):166. doi: 10.3758/s13428-026-03008-x (PMC13171683; doi:10.3758/s13428-026-03008-x)
Supplement: Supplementary file 1 — Supplementary file1 (PDF 2853 KB) [file 13428_2026_3008_MOESM1_ESM.pdf]

Appendix

Random slope results

Rejections rates

For both random intercept and random slope models, Figure A1 presents the Type I error rates across replications, grouped by the missingness mechanism. The results are broadly consistent with those observed for the random intercept model. Notably, the Random Forest imputation method without PMM fails to maintain the significance level across all variables, regardless of the combination of missingness rate, missingness mechanism, or cluster size. This issue is particularly pronounced for level-2 variables, where Type I error rates are significantly higher than 0.05. For the MCAR mechanism, Type I errors exceed 0.20 in some cases. Consequently, we exclude Random Forest without PMM from the power analysis.

For scenarios with 50% missingness, even the Random Forest method with PMM exceeds the acceptable significance level. While MAR with a cluster size of 25 shows that the introduction of PMM can reduce Type I error rates below 0.05, in other scenarios, the Random Forest with PMM and dummy variables still produces Type I error rates above the acceptable threshold. In contrast, the boosting methods and MICE consistently maintain the significance level, demonstrating their robustness in this context.

In Figure A2, the rejection rates for the random intercept and random slope models are presented. The primary differences in the comparison of the imputation methods arise again with varying missing rates. For 10% missingness, all methods exhibit similar rejection rates for the level-1 variables. However, for the level-2 variables, MICE clearly outperforms the other imputation methods, with its advantage being more pronounced when the cluster size is 25. For a cluster size of 50, MICE remains the best-performing method, but the tree-based methods show performance closer to that of MICE.

Similar to the random intercept model, a missingness rate of 30% also leads to mixed results for the random intercept and random slope models. For the level-1 variables, `mixgb` (with and without dummies) outperforms all other imputation methods, with a slight advantage for the version using dummies. In contrast, for the level-2 variables, MICE clearly emerges as the best option, with only minor differences observed among the tree-based methods.

At 50% missingness, the advantage of `mixgb` (with and without dummies) for level-1 variables increases significantly under MCAR. For MAR, the boosting method with dummies remains the top choice for level-1 variables, though the performance gap to MICE is smaller. The largest rejection rates for level-2 variables at 50% missingness are observed with the Random Forest method without dummies; however, these results are unreliable as this method leads to an inflated type I error rate.

Regardless of the missing data mechanism or cluster size, `mixgb` with dummies generally performs slightly better than `mixgb` without dummies. Overall, the level-2 rejection rates for MICE are comparable to those of the boosting methods. However, given the advantages for the level-1 variables, `mixgb` with dummies should be preferred over MICE at a 50% missingness rate.

Coefficient bias

Figure A3 presents the coefficient bias and relative bias for each method under the random intercept and random slope model. The first plot shows the bias for the true zero coefficients at level-1 and level-2, while the second plot shows the relative bias (adjusted for effect size) for the non-zero coefficients. Overall, the pattern is quite similar to the previous figure, with a few notable differences.

For the true zero coefficients at both levels, the bias values remain close to zero, even with increasing missingness rates or decreasing cluster sizes. This suggests that the imputation methods do not introduce any significant bias for coefficients that are expected to be zero.

The more interesting observation concerns the relative bias for the non-zero coefficients. Similar to the previous figure, all imputation methods induce a negative bias, but the magnitude of this bias varies across methods and scenarios. Notably, MICE shows a relatively large negative bias for the intercept when the cluster size is 25 and the missingness rate is 50%. This stands in contrast to the other methods, which do not show a similarly pronounced bias for the intercept.

The relative bias for the slope coefficients aligns with the findings from the previous analysis. `mixgb` (both with and without boosting) exhibits a relatively high bias for level-2 variables, especially when compared to MICE, which shows the lowest bias for the level-2 coefficients, regardless of missingness rate or cluster size. For level-1 variables, MICE shows a larger bias than the boosting methods, and this difference increases as the missingness rate rises.

In general, the Random Forest methods show a relatively large bias for both level-1 and level-2 variables across the different missingness rates and cluster sizes. For the MCAR missingness mechanism, the results remain largely consistent, with only minor differences compared to the MAR scenario.

Standard deviation

In Figure A4 we present the ratios of standard deviation of the parameter estimation on the imputed dataset and on the complete dataset. The general tendencies of the analysis of the random intercept model remain true. A difference to the random intercept model is that for level-1 features with a small effect MICE and `ranger` underestimate the true standard deviation. For the larger level-1 effect we can see the usual overestimation of standard deviation.

For level-2 variables, SD inflation is more pronounced, especially at a 50% missing rate. With a cluster size of 25, the SDs for MICE are sometimes more than ten times larger than in the complete data set. This explains the relatively low power of MICE despite its smaller negative bias. `missRanger` with PMM obtains the best SD at a 50% missing rate, while MICE is closer to the tree-based methods at a cluster size of 50. At a 10% missing rate, MICE outperforms the alternatives.

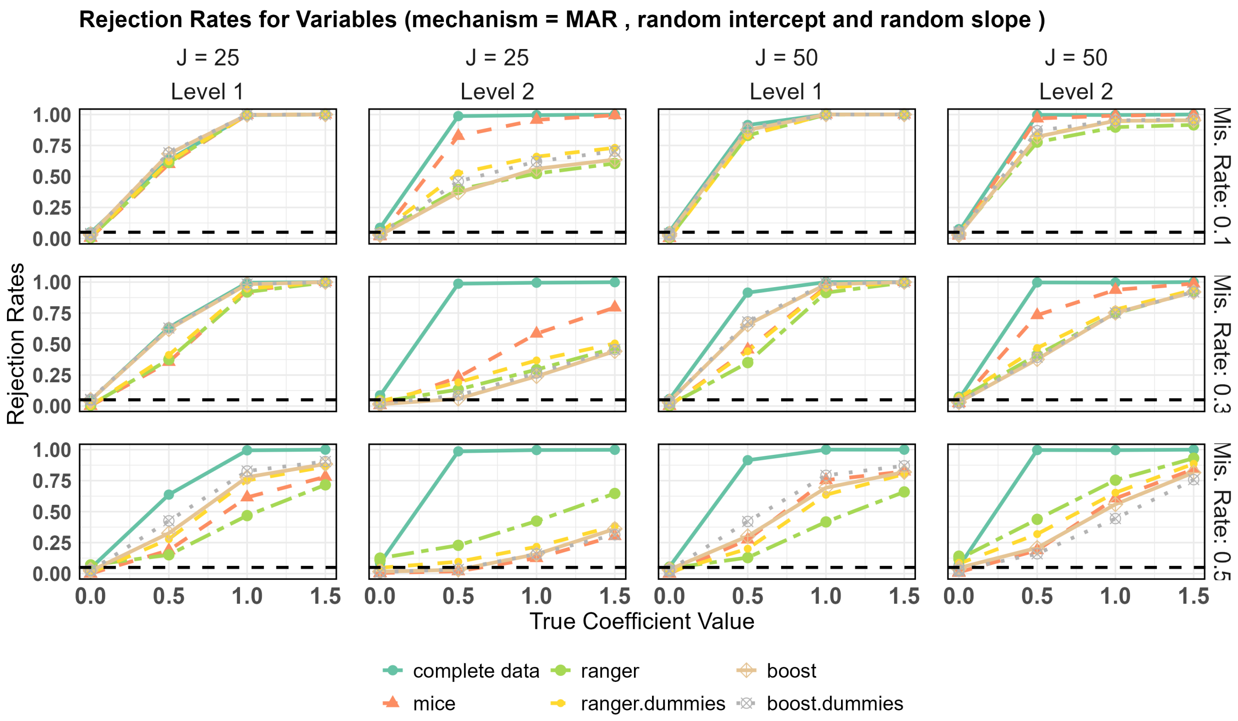

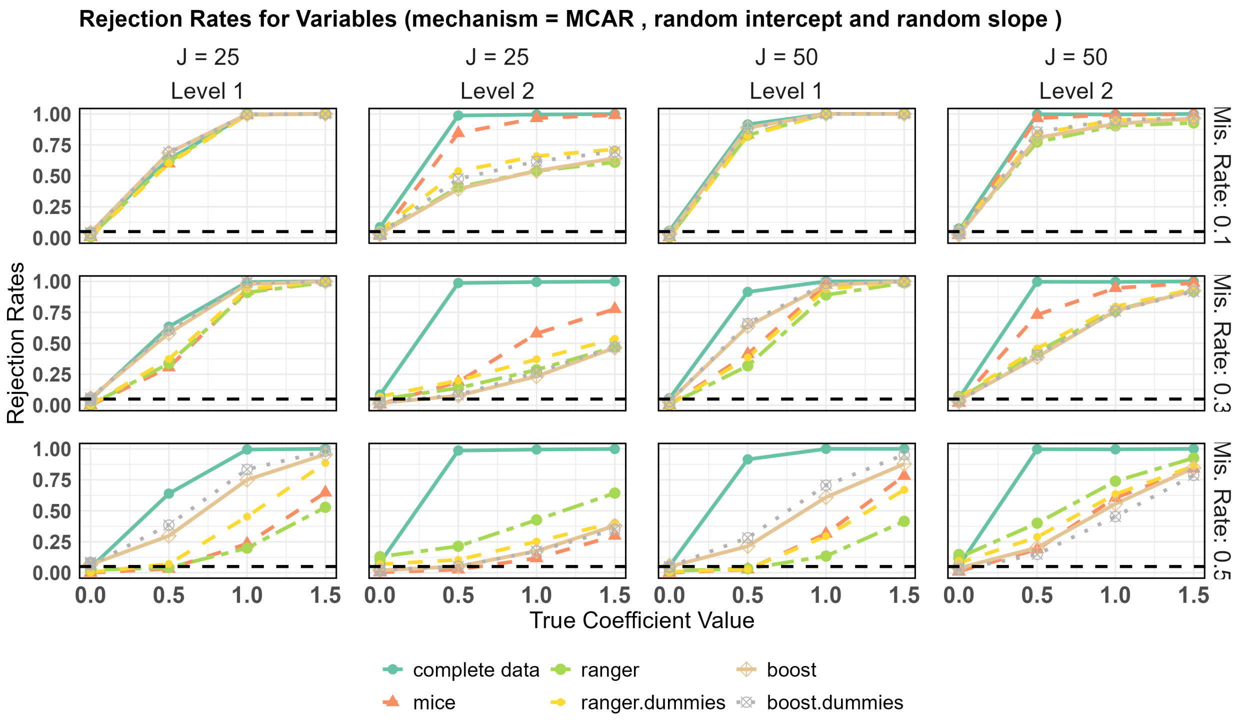

Figure A1

Type I Error for random intercept and random slope designs.

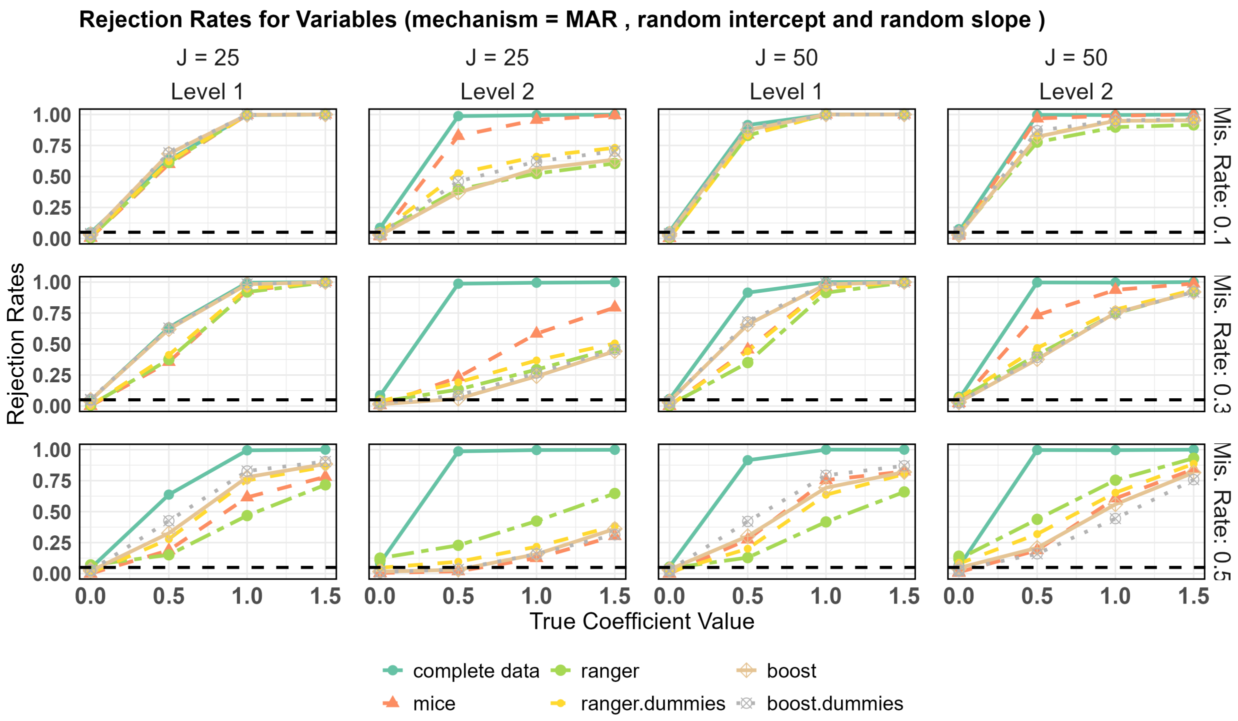

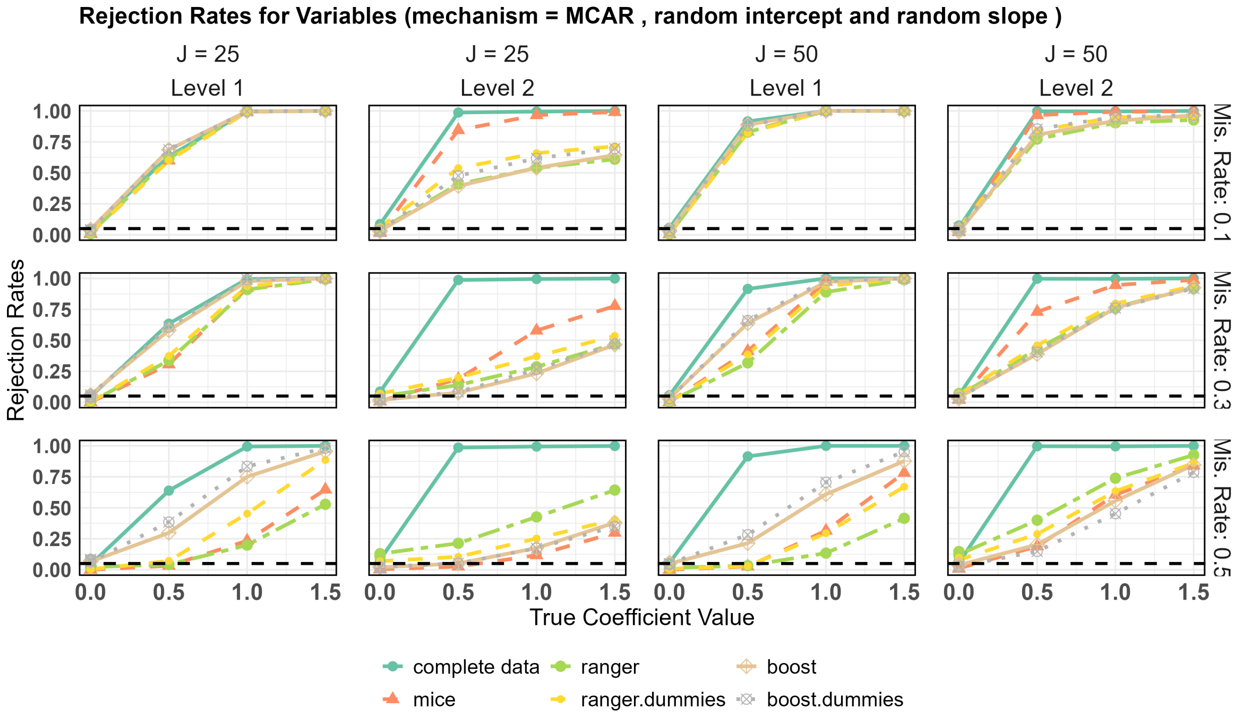

Figure A2

Power for random intercept and random slope designs.

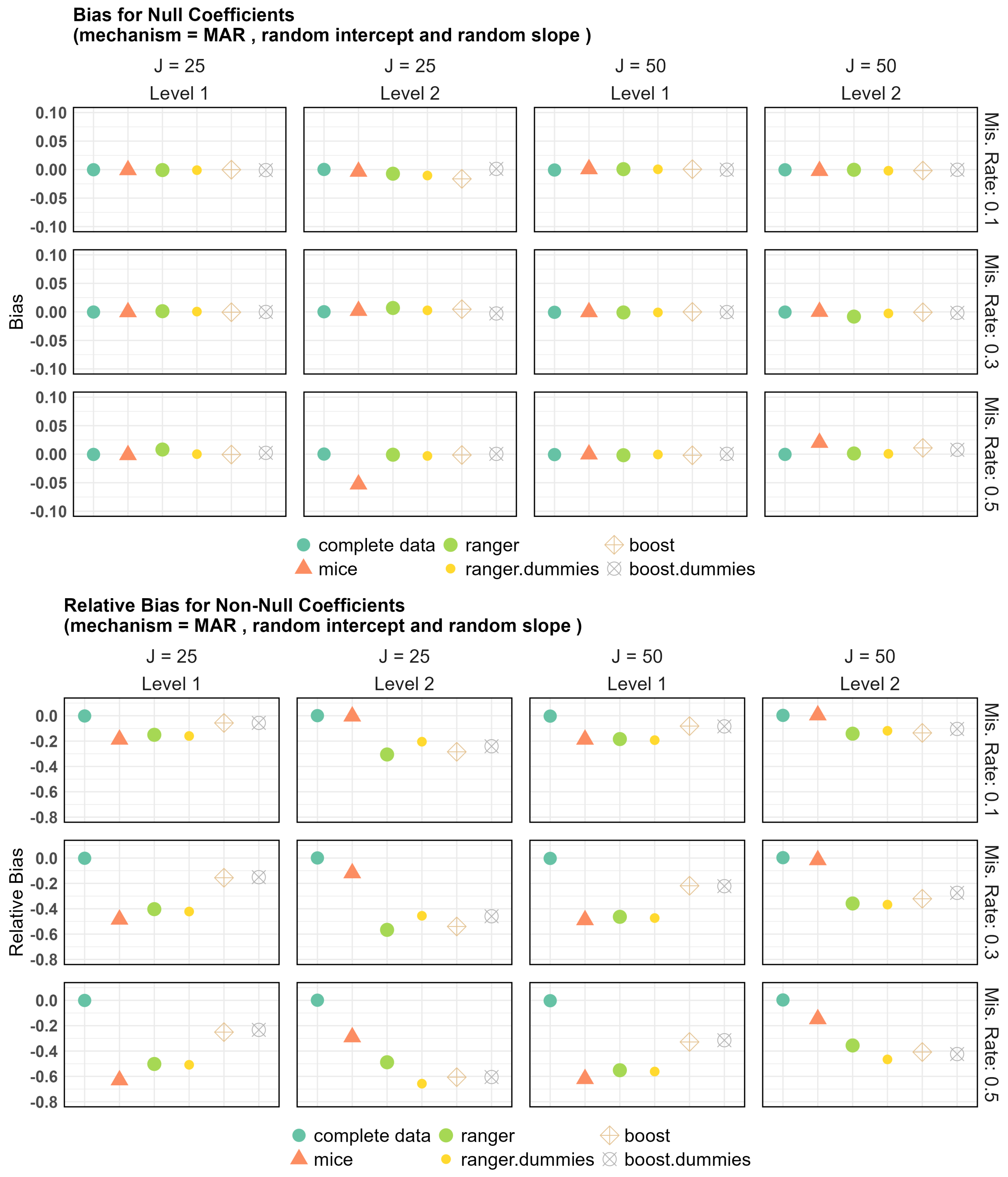

Figure A3

Bias for the true zero coefficients and Relative Bias for the non-zero coefficients.

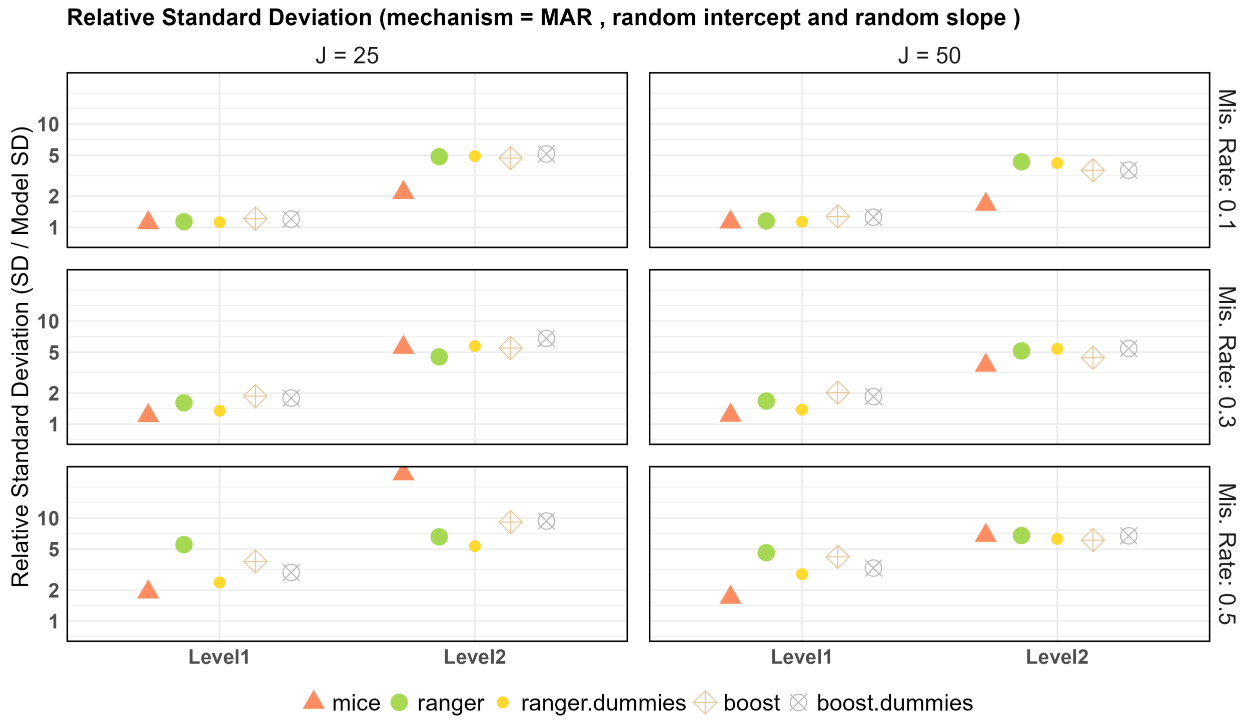

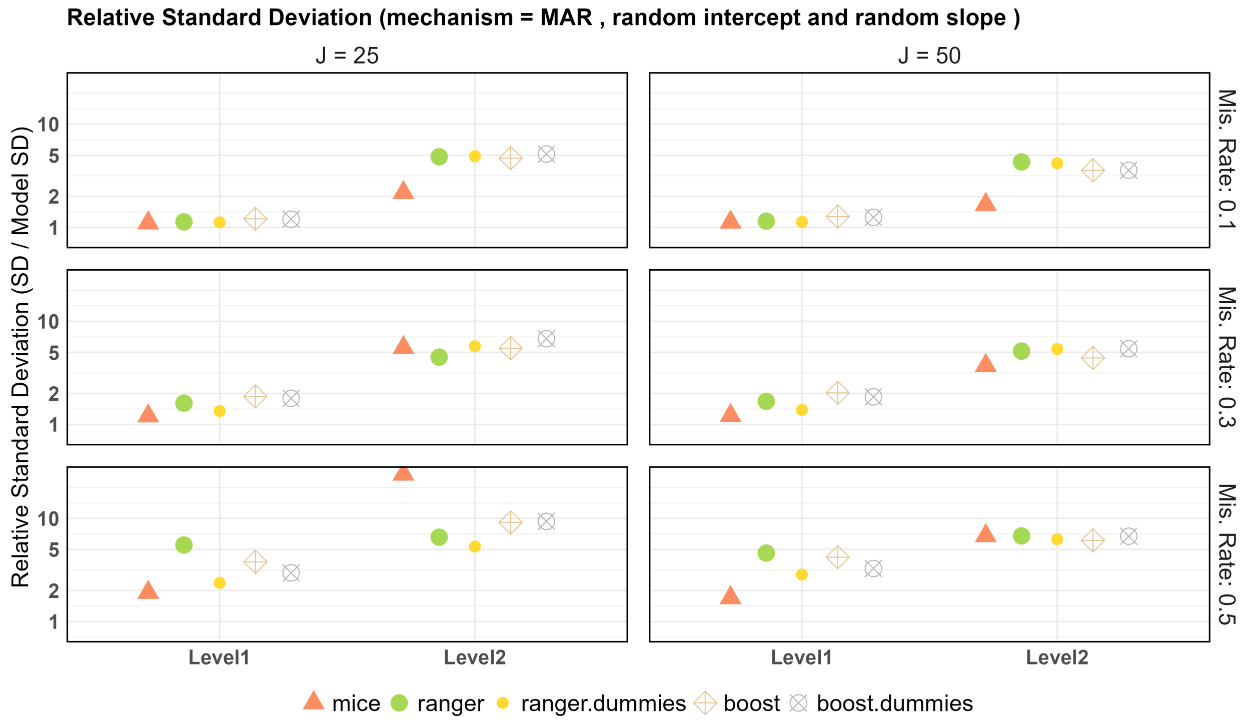

Figure A4

Ratio of standard deviation (imputed data vs. complete data) for the random intercept and random slope model.

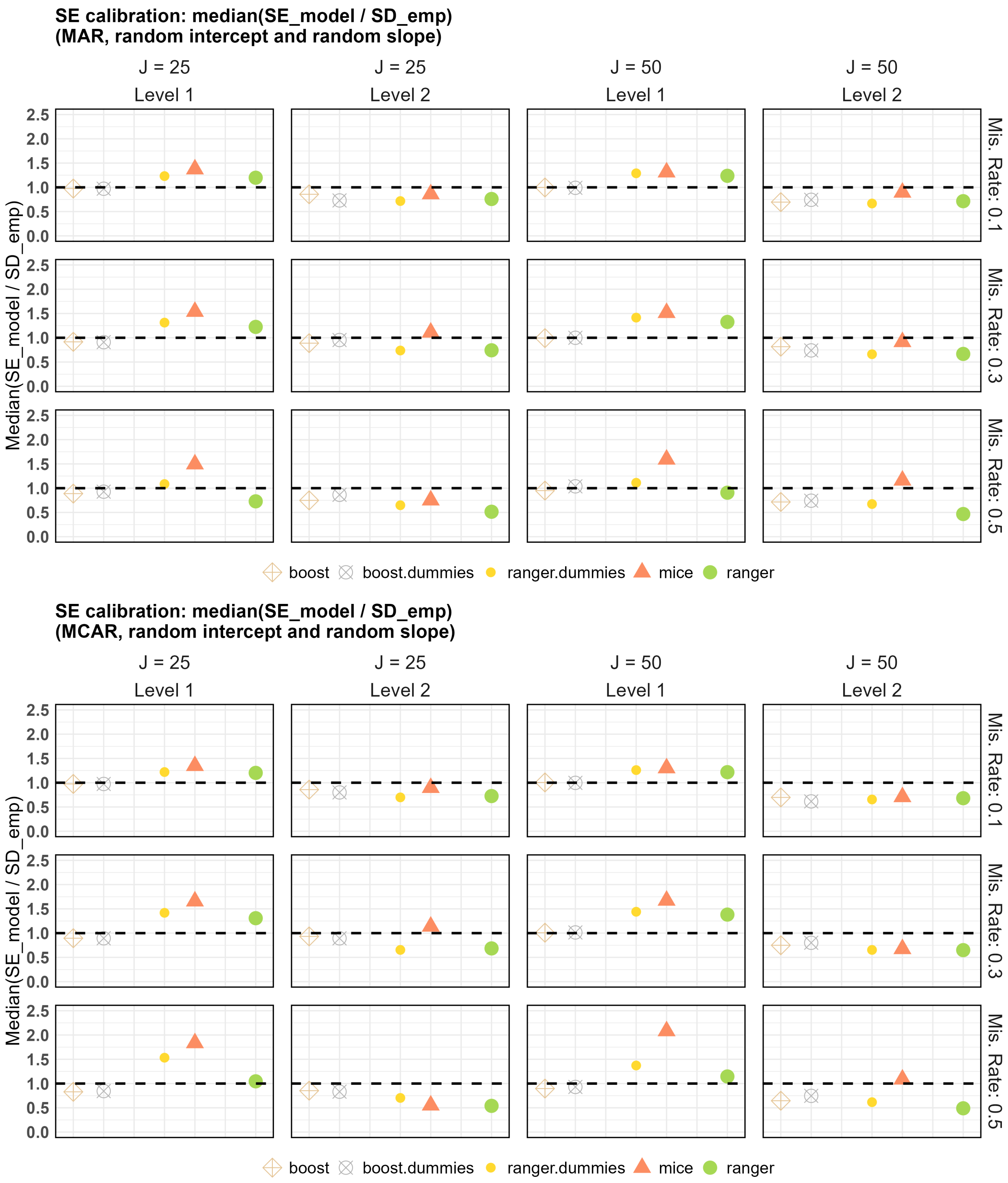

Figure A5

Calibration of model-based standard errors for the random intercept and random slope model. Shown are ratios of average model-based standard errors to empirical standard deviations across replications.
